# Epigenetics and male reproduction: the consequences of paternal lifestyle on fertility, embryo development, and children lifetime health

**DOI:** 10.1186/s13148-015-0155-4

**Published:** 2015-11-11

**Authors:** Liborio Stuppia, Marica Franzago, Patrizia Ballerini, Valentina Gatta, Ivana Antonucci

**Affiliations:** Laboratory of Molecular Genetics, Department of Psychological, Health and Territorial Sciences, School of Medicine and Health Sciences, “G. d’Annunzio University” Chieti-Pescara, Via dei Vestini 31, 66013 Chieti, Italy; Laboratory of Pharmacogenetics, Department of Psychological, Health and Territorial Sciences, School of Medicine and Health Sciences, “G. d’Annunzio University” Chieti-Pescara, Via dei Vestini 31, 66013 Chieti, Italy; Ce.S.I.-MeT, “G. d’Annunzio” University, Chieti-Pescara, Via dei Vestini 31, 66013 Chieti, Italy

**Keywords:** Male infertility, Gametogenesis, Epigenetics, DNA methylation, Transgenerational effect

## Abstract

The correlation between epigenetics and human reproduction represents a very interesting field of study, mainly due to the possible transgenerational effects related to epigenetic modifications of male and female gametes. In the present review, we focused our attention to the role played by epigenetics on male reproduction, evidencing at least four different levels at which sperm epigenetic modifications could affect reproduction: (1) spermatogenesis failure; (2) embryo development; (3) outcome of assisted reproduction technique (ART) protocols, mainly as concerning genomic imprinting; and (4) long-term effects during the offspring lifetime. The environmental agents responsible for epigenetic modifications are also examined, suggesting that the control of paternal lifestyle prior to conception could represent in the next future a novel hot topic in the management of human reproduction.

## Background

*You shall not bow down to them or worship them;for I, the Lord your God, am a jealous God,punishing the children for the sin of the parentsto the third and fourth generation of those who hate me*

*(Esodus, 20.5)*

Infertility represents a growing emergency in western countries, affecting about one out of seven couples who attempt to generate a child. In 2010, an estimated 48.5 million couples worldwide were infertile, against 42 million in 1990 [1]. In about 50 % of the cases, this condition is ascribable to the male partner, mainly due to a failure in the spermatogenesis process causing azoospermia or oligozoospermia at the sperm count [2]. Despite the large number of tools available for the identification of the pathogenesis of male infertility, in many cases, no specific cause is detected and no personalized therapeutic protocol can be established. A large number of studies have investigated in the last decades the presence of genetic alterations responsible for the failure of spermatogenesis, which are nevertheless identified only in 15–30 % of infertile males, even when stringent selection criteria are used [3, 4]. Despite the identification of several rare genetic variants associated to disruption of spermatogenesis, so far, the only two categories of genetic alterations responsible for a significant portion of cases of male infertility, and thus commonly tested in the clinical practice, are represented by chromosomal alterations and Yq microdeletions [5–10]. The presence of other genetic mechanisms, such as partial Yq microdeletions [11–14], specific Y-chromosome haplogroups [15–18], and polymorphism in genes related to mitochondrial function [19–21] as risk factors for infertility have been suggested, but with inconclusive results. More recently, the presence of X-linked copy number variants (CNVs) in infertile males has been reported by several studies [22, 23], but the overall incidence of these variants accounts only for a limited portion of all cases.

In recent years, great interest has been raised by the novel acquisitions on the epigenetic mechanisms of regulation of gene expression. Epigenetics can be defined as the study of mitotically or meiotically heritable modifications in the function of specific genes not related to modification in the DNA sequence [24]. This novel field of study has obtained large relevance also in the world of mass media, usually transmitting the “take-home message” that human destiny is not written inside genes, since environmental agents or experiences can influence human heredity [25]. As a matter of fact, the interaction between genes and environment in the determination of human phenotypes is very well known from many years, but the real novelties provided by the studies on epigenetics are that (1) environmental agents can modify the expression of specific genes without changing their sequence or copy number, and (2) these modifications can be transmitted to the offspring, so that either rare congenital diseases or the susceptibility to common diseases appearing during the lifetime can be the result of a gene-environment interaction that occurred in one parent of a subject, not in the subject himself. In this view, epigenetic studies represent a breakthrough in the field of human reproduction. In fact, since epigenetic modifications can be transmitted to the offspring, they obviously involve germ cells, and in some cases, they could affect gametogenesis as well as the embryo development, thus representing a potential cause of infertility of the couple. Moreover, since epigenetic alterations do not induce modification in the gene sequence or copy number, they could account for at least a portion of cases of male infertility in which no genetic abnormalities are detected using the conventional techniques of genetic analysis.

Several studies have investigated in the last years the role played by epigenetic modification in male gametogenesis and in male infertility. The aim of this review is to analyze the state-of-art of this field of research in order to give an answer to the following questions. (1) Can epigenetic mechanisms be related to the quality of the spermatogenesis process? (2) Can sperm epigenetic alterations affect embryo development? (3) Is there a relationship between sperm epigenetic modifications and outcome of assisted reproduction technique (ART) procedure? (4) Which environmental agents can be responsible for epigenetic modifications of sperm DNA?

### Molecular basis of epigenetics

The main epigenetic mechanisms of gene expression regulation are represented by DNA methylation, histone modifications, and small, non-coding RNAs.

#### DNA methylation

**Fig. 1:**
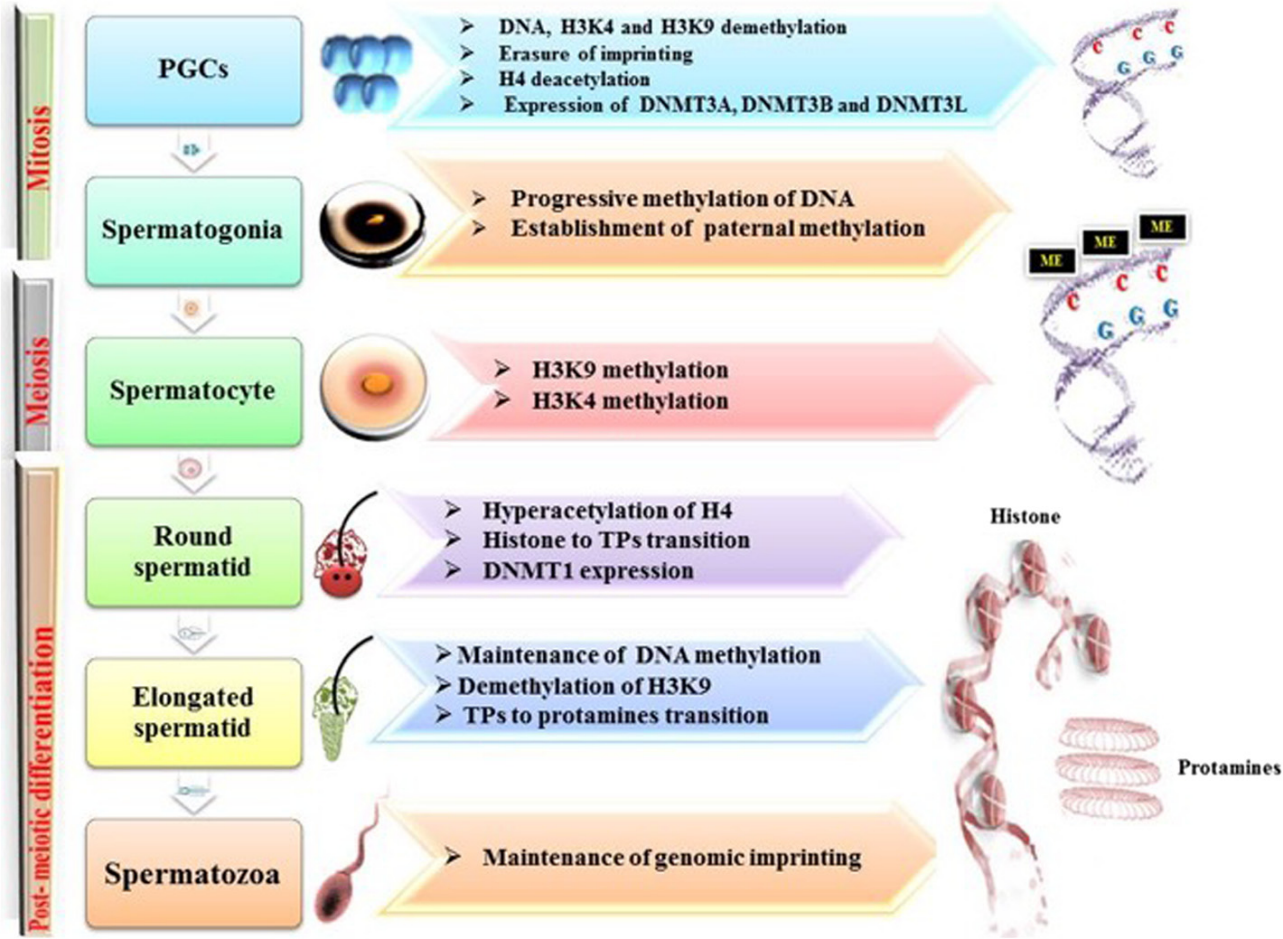
Epigenetic modifications during spermatogenesis. During the different steps of spermatogenesis, several epigenetic modifications involving DNA methylations and histone modifications occur. (1) PGCs undergo a process of demethylation involving DNA (with erasure of genomic imprinting) and histones (namely, K4 and K9 residues of H3). Also, a process of H4 deacetylation is present. DNMT3A, DNMT3B, and DNMT3L are expressed at this time. (2) In spermatogonia, a progressive DNA methylation occurs, with establishment of paternal methylation. (3) In spermatocytes, H3K9 and H3K4 methylation is observed. (4) In round spermatids, H4 becomes hyperacetylated, DNMT1 is expressed, and the transition from histones to TPs occurs. (5) Elongated spermatids show a maintenance of DNA methylation, together with H3K9 demethylation. The transition from TPs to protamines occurs at this step. (6) In spermatozoa, the genomic imprinting is maintained

In mammalians, DNA methylation occurs at the 5′- position of cytosine residues, mainly within CpG dinucleotides, 60–80 % of which are methylated within the promoter regions of genes [24]. Methylation of CpG dinucleotides within the promoter regions leads to the silencing of transcription process, mediated by modifications in the condensation status of the chromatin. The process of DNA methylation is catalyzed by enzymes known as DNA methyltransferases (DNMTs), which can be classified in “de novo” DNMTs (which methylate specific chromosomal sequences during early embryogenesis), and maintenance methyltransferases (DNMT1), faithfully restoring the methylation patterns after each DNA replication cycle [26]. The process of DNA methylation is closely related with gametogenesis, since primordial germ cells (PGCs), when entering the developing gonad, undergo a process of deep decrease of DNA methylation, which will be subsequently restored in the prenatal life in males and during post-natal follicle development in females (Fig. 1) [27].

Cytosine methylation can occur also in non-CpG sites (CpA > CpT > CpC), although the significance of these variants is still unknown. Methylation at non-CpG sites was previously considered to be largely present in the brain, embryonic stem cells (ESCs), induced pluripotent stem cells, and in oocytes [28–32]. However, Ichiyanagi et al. observed methylation at non-CpG sites in male germ cells as well, demonstrating that the level of the non-CpG methylation is higher in prospermatogonia and decreases along with mitotic division. In addition, this study also suggested the absence of a template-dependent mechanism for copying non-CpG methylation in prospermatogonia [33].

Another kind of epigenetic DNA modification is represented by 5-hydroxymethylcytosine (5hmC), an intermediate of DNA demethylation with important regulatory functions in various biological and pathological processes in the mammalian genome such as transcriptional regulation, DNA methylation regulation, and tumorigenesis [34]. Gan et al. showed that the highly ordered 5hmC alterations are critical for the differentiation of spermatogenic cells in the mouse [35]. Recently, Wang et al. for the first time compared 5-hydroxymethylcytosine profiles in normal, abnormal, and globozoospermia sperms, identifying 6664, 9029, and 6318 genes containing 5hmC, respectively [36]. In addition, since some 5hmC-containing genes are significantly involved in spermatogenesis, sperm motility, and morphology, the authors suggested that the 5hmC distribution differences may contribute to the sperm phenotype [36].

DNA methylation was originally investigated by Southern blot and methylation-sensitive restriction endonuclease digestion followed by PCR amplification [37]. Nowadays, gene-specific techniques are available including bisulfite sequencing, combined bisulfite restriction analysis (COBRA) [38], MethyLight [39], and bisulfite pyrosequencing [40], all based on bisulfite conversion of cytosine residue to uracil, leaving 5-methylcytosine residue unaffected [41], detectable by DNA sequencing. All the abovementioned techniques are sensitive, specific, and relatively inexpensive but not suitable for analysis of the whole genome, which includes about 28 million CpGs.

The next-generation sequencing (NGS) approach represents a new powerful tool for the study of human DNA methylome [42]. The NGS DNA methylation procedures are based on three main steps: digestion of genomic DNA with methyl-sensitive restriction enzymes, affinity-based enrichment of methylated DNA fragments, and chemical conversion methods [43, 44].

Another large-scale approach is represented by whole-genome bisulfite sequencing (WGBS), which is able to analyze DNA methylation profiles of whole genomes at single-base resolution [45]. Recently, several studies have reported WGBS accurate data on methylome both of human embryonic stem cells [46] and induced pluripotent stem cells [47].

#### Histone modifications

Modifications of histone tails represent other epigenetic chromatin marks critical for transcriptional regulation. Post-translational modifications of histone tails include methylation, acetylation, phosphorylation, ubiquitination, ribosylation, and sumoylation. Histone marks are dynamic process, since histone modifications can be easily induced and removed by a wide range of enzymes [48]. The most relevant histone change is represented by acetylation at lysine residues on the amino-terminal tail domains, whose correct levels are maintained by the combined action of two enzymes known as histone acetyl transferase (HAT) and histone deacetylase (HDAC). Generally, acetylation reduces the affinity of histones for DNA, making genes functionally active; on the other hand, histone deacetylation leads to chromatin condensation, making genes transcriptionally inactive. Conversely, histone methylation is a key regulator for both activation and inactivation of transcription. For example, lysine 4 of histone H3 (H3-K4) methylation is linked to gene expression, while H3K9 and H3K27 di- and tri-methylation is associated with gene silencing [49, 50].

Histone H3T is the testis-specific H3 variant in mammals. Tachiwana et al. showed that H3T nucleosomes can be assembled by Nap2 chaperone and that this structure is significantly unstable as compared to the conventional H3.1-containing nucleosomes [51, 52]. Furthermore, these authors suggested that the unstable H3T-containing nucleosome structure can influence the chromatin re-organization essential for spermatogenesis [52].

Histone modifications were originally investigated by Western blot with specific antibodies to modified histones, but nowadays, they are mainly studied by mass-spectrometry-based proteomic technologies [53]. Recently, NGS technologies have been developed able to analyze whole-genome histone modifications. These techniques are based on chromatin immunoprecipitation followed by sequencing maps of the genome-wide binding pattern of chromatin-associated proteins, which includes modified histones (ChIP-seq) [44, 54].

#### Small non-coding RNAs

A third mechanism of epigenetic regulation of gene activity is represented by small RNAs and large intergenic non-coding RNAs (lincRNAs). Small RNAs, not encoding proteins, are located in the nucleus of spermatozoa and play an essential role as epigenetic modifiers both in recognizing and preserving the DNA that remains bound to histones during the transition to protamine in spermiogenesis and in early embryonic development [55]. On the other hand, lincRNAs act at the level of chromatin allowing to select the histone modification enzymes, such as in the case of lincRNAs HOTAIR which acts as a scaffold for PRC2 and LSD1 enzymes to regulate lysine 27 methylation and lysine 4 demethylation in H3 histone [56].

Small non-coding RNAs are generally identified by RT-PCR analysis, in situ hybridization, or small RNA sequencing studies. Furthermore, the recent development of microarray technologies has permitted the global analysis of spermatozoal microRNAs (miRNAs) evidencing different expression profiles between fertile and infertile men [57, 58].

### Genomic imprinting

A specific feature of epigenetic control of gene function is represented by genomic imprinting, a process leading to the expression of a specific set of genes (about 70–80, the majority of which clustered in 16 specific chromosomal regions) based on their maternal or paternal origin [59]. Genomic imprinting plays a key role in the regulation of resource acquisition by the offspring from the mother during prenatal and early postnatal life. Paternally and maternally imprinted genes play different roles in this mechanism, being many paternally expressed alleles able to increase resource transfer to the child, which is on the other side reduced by maternally expressed genes (“parental conflict hypothesis”) [60]. The correct balance between the activity of maternally and paternally imprinted genes can be disrupted by different mechanisms, such as chromosome deletions, uniparental disomy (UPD), or alterations in the imprinting center. In human, alterations of the process of genomic imprinting cause several congenital diseases mainly involving fetal growth (e.g., Beckwith–Wiedemann syndrome, Russell–Silver syndrome), hormone systems after birth (e.g., Albright hereditary osteodystrophy, pseudohypoparathyroidism 1A, transient neonatal diabetes mellitus), or behavior (e.g., Prader–Willi syndrome, Angelman syndrome) [60]. Moreover, imprinting alterations have been suggested as responsible for intrauterine growth restriction, in turn associated with an increased risk of cardiovascular disease, diabetes, and mental defects later in life [60, 61].

The discovery of the role played by epigenetic modifications on the function of paternal genome has prompted novel attention on the function of sperm DNA during embryo development. In fact, sperms have been historically viewed as specialized cells with the unique function of delivering the 23 paternal chromosomes to the oocyte, considered as the only gamete playing an active role in driving embryo development, thanks to its availability of cellular organelles, RNAs, and cellular machinery. Actually, the role played by the male gamete in embryo development appears to be more relevant than previously hypothesized. In this view, a full knowledge of the epigenetics of sperm could provide novel information about germ cell biology, paternal effects on embryogenesis, and the pluripotency of embryonic stem cells [62].

### Epigenetics and spermatogenesis

#### The process of spermatogenesis

The formation of a mature sperm requires different processes, namely (1) mitotic proliferation of spermatogonia; (2) meiotic divisions; and (3) morphological differentiation of sperm precursors (spermiogenesis), leading to the generation of highly specialized cells characterized by the presence of a head, an intermediate portion, and a flagellum. Such a specific organization of the male germ cells is necessary to allow sperms to traverse a potentially hostile female reproductive tract, penetrate the cumulus oophorus and the zona pellucida, penetrate the oocyte, and finally complete multiple post-penetration events [62, 63]*.* During fetal life, spermatogenesis begins in the wall of the seminiferous tubules from undifferentiated diploid cells known as spermatogonia, which undergo several mitotic divisions in order to increase the pool of available precursors of germ cells. At puberty, some spermatogonia are transformed in type I spermatocytes, which undergo the first meiotic division producing haploid type II spermatocytes. A second meiotic division occurs in these cells, originating haploid spermatids. The last phase of spermatogenesis is represented by spermiogenesis, characterized by a morphological and structural transformation complex process of the round spermatid. This step, occurring without further cell division, leads to the production of mature sperm, characterized by the differentiation of the flagellum and the acrosome, essential prerequisites for sperm motility and fertilization capacity.

#### Histone–protamine replacement as the main epigenetic change in sperms

In addition to the typical morphology and motility, sperms are characterized also by a highly organized chromatin structure. In fact, sperm chromatin during spermiogenesis undergoes further condensation, due to the replacement of 90–95 % of the histones with one or more sperm-specific basic proteins, known as protamines [64]. This modification induces the formation of disulfide bonds (SS) that confer extreme stability to the core of the sperm nucleus, producing a number of relevant effects, such as improvement of sperm motility, protection from oxidative stress and toxic agents present within female reproductive tract, and block of the transcriptional activity of the sperm DNA [65]. The complex mechanism of histone–protamine transition is a finely regulated multi-step process. In the first step, the histones in round spermatids are replaced by a heterogeneous group of nuclear proteins (transition proteins (TP)), as the result of histone hyperacetylation [66] (Fig. 1). The second step takes place in elongating spermatids, determining the replacement of TP1 and TP2 with protamines [62, 67] (Fig. 1). Protamines have different functions: they allow the compaction of the nucleus ensuring the genetic integrity of the sperm and play an important role in epigenetic imprinting [62]. Mature spermatid nuclei present two types of protamines: the P1 protamine and the P2 family of protamines, constituted by P2 (the most abundant), P3, and P4 members. P1/P2 ratio appears to be critical for male fertility [68, 69]. In fact, the P1/P2 ratio, which in fertile males is close to 1 (range 0.8–1.2), is altered in infertile patients [69, 70]. Patients with a P1/P2 ratio <0.8 present inadequate DNA condensation and important alterations in sperm parameters, such as motility, counts, and structure [68–71]. Moreover, Aoki et al. demonstrated that low P1/P2 ratios are also associated with an increased DNA fragmentation, which is also inversely correlated with global sperm P1 and P2 concentrations, suggesting a protective role of the protamines against sperm DNA damage [72]. There is also evidence that subfertility can be correlated with an excess of protamine P2 precursors (pre-P2), determined by an alteration of the process leading to the mature protamine P2 formation [68, 71, 73, 74].

#### DNA methylation and histone modifications during spermatogenesis

Various and specific epigenetic marks are required during male gametogenesis for proper maturation of gametes. In fact, before meiosis, the first epigenetic events take place in the form of progressive demethylation–remethylation of DNA. During meiosis, DNMT3A, DNMT3B, and cofactor DNMT3L activity regulates the levels of de novo DNA methylation, completing this process after birth at the stage of pachytene spermatocyte [75]. Subsequently, the methylation profile is maintained by DNMT1 activity. In addition to the above-described processes, also histone modifications (methylation and acetylation) occur, which modify DNA accessibility to transcription factors (Fig. 1). In fact, specific enzymes such as histone methyltransferase (HMT) and histone demethylase (HDM) regulate lysine 9 of histone H3 (H3-K9) and lysine 4 of histone H3 (H3-K4) methylation patterns. Generally, histone H3-K9 methylation is high in meiosis but is removed at the end of this process, promoting gene activation, whereas histone H3-K4 methylation, which decreases during meiosis, is associated to DNA silencing [50] (Fig. 1). In addition, during spermatogenesis, several enzymes, such as HAT and HDAC, regulate the processes of acetylation and deacetylation of H3 and H4 lysine residues. During spermiogenesis, hyperacetylation of H4 plays a crucial role for correct histone to protamine transition and allows nucleosome disassembly in elongating spermatids [66, 76] (Fig. 1).

#### Epigenetic alterations and spermatogenesis disruption

The above-described epigenetic marks in germ line genes play a key role in the proper spermatogenesis processes, and several studies have demonstrated that aberrant epigenetic modification of genes expressed in the testes are associated with male infertility. Navarro-Costa et al. for the first time hypothesized that one of the factors of male gametogenic defects could be represented by epigenetic alterations of specific genes, evidencing increased methylation defects of the germ line regulator *DAZL* gene in different quality-fractioned sperm populations of oligoasthenoteratozoospermic (OAT) patients as compared to normozoospermic (NZ) men [77]. On the contrary, no variation in the methylation state of the *DAZL* gene promoter between NZ e OAT men was observed. This study also highlighted the existence of homogeneous *DAZL* methylation levels when comparing the normal sperm-enriched fractions of NZ men. This evidence has been subsequently confirmed on a larger scale by Krausz et al. who detected no differences in the DNA methylation status of several genes in the different sperm subpopulations of normozoospermic individuals [78]. Subsequently, other studies have identified more genes whose epigenetic modifications are related to alteration of both semen parameters and fertility maintenance. Hammoud et al., by analyzing seven imprinted loci (*LIT1*, *MEST*, *SNRPN*, *PLAGL1*, *PEG3*, *H19*, and *IGF2*), correlated alterations in the DNA methylation pattern of oligozoospermic patients with abnormal protamine levels [70]. Interestingly, alterations in the mRNA levels of the genes involved in the histone–protamine transition have been evidenced also by studies carried out by investigating the global testis transcriptome of normal and oligozoospermic patients by microarray analysis [79]. Other studies have demonstrated that DNA hypermethylation of the promoter of several genes (such as *MTHFR*, *PAX8*, *NTF3*, *SFN*, *HRAS*, *RASGFR1*, *GTL2*, *PLAG1*, *D1RAS3*, *MEST*, *KCNQ1*, *LT1*, *SNRPN* and others) plays a critical role in male infertility, being associated to alterations of sperm concentration, motility, and morphology, while hypomethylation of the *IGF2/H19* imprinting control region 1 (ICR1) has been detected in patients with low concentration and sperm motility as compared to normozoospermic controls [70, 80–86].

Due to the increased knowledge about the epigenetic alterations occurring in sperm DNA of infertile patients, it has become clear that specific errors in the processes of epigenetic control may occur during each stage of spermatogenesis, adversely affecting male fertility and embryonic development [49]. In fact, epigenetic alterations occurring in mitosis can affect the expression of specific genes involved in the first steps of spermatogenesis, leading to a decreased efficiency of the process. When the meiotic stage is involved, these alterations can induce double-strand breaks or chromosomal non-disjunction. Finally, during spermiogenesis, epigenetic alterations can involve histone-to-protamine transition and histone removal and degradation, inducing protamine replacement errors [49]. Taken together, all these evidences suggest that different features of male infertility, such as alterations in sperm count or morphology, DNA fragmentation, chromosomal aneuploidies, and alterations in the chromatin package, could be all related to epigenetic mechanisms occurring at different stages of spermatogenesis.

### Epigenetics and embryo development

DNA methylation and histone modifications play a crucial role in the process of genome reprogramming during early embryogenesis [87–89]. Preimplantation embryo development is a dynamic process characterized by deep gene expression profile change and modifications in the histone and chromatin organization [87, 90–92]. After fertilization, the genome of paternal and maternal origin is subjected to a process of reprogramming: at first, the male pronucleus is demethylated [93, 94], and after the formation of the zygote, the chromosomes of both parents are demethylated by a passive mechanism erasing most parts of the methylation marks except those involved in the process of genomic imprinting [95]. The methylation of imprinted genes is erased only in PGC, cells of epiblast that give rise to male and female gametes. Here, an extraordinary epigenetic regulation occurs in the early stages of embryonic development, when the methylation is erased and specific genes of pluripotency (*OCT4* and *NANOG*) are expressed. De novo methylation starts in the inner cell mass of the blastocyst and the levels of methylated DNA increase in primitive ectoderm, while methylation is inhibited in the trophoblast and in the primitive endoderm [96, 97]. This alternation of demethylation and remethylation can be explained by the necessity during preimplantation to activate zygotic genes essential for early development, while de novo methylation could establish a state of global silencing in order to suppress retrotransposons [87]. In post-implantation embryos, the maintenance of DNA methylation is crucial for embryonic development. In female embryos, X inactivation, due to increased expression and accumulation of Xist RNA, occurs [98]. The X chromosome is inactivated to compensate the number of X-linked genes in males and females, and the process of inactivation takes place after the implantation of female embryos or during the process of differentiation of ESCs [99, 100]. A reactivation of the X chromosome occurs in the inner cell mass of the blastocyst and in the epiblast, followed by a random inactivation, a biological process in which both X chromosomes have the same probability of being inactivated [101, 102].

A first relevant role played by paternal genome in the above-described process is represented by genomic imprinting. In fact, at least three paternally imprinted genes, *H19-IGF2*, *RASGRF*, and *DLK1-GTL2* are considered among the most relevant for embryonic development and placentation [103–105]. However, genomic imprinting does not appear to represent the only mechanism of paternal control on embryo development. As a matter of fact, it has been demonstrated that the presence of modified histones in the spermatozoa could represent a potential paternal contribution in epigenetic reprogramming of the zygote regardless of the imprinting process [106–108]. In fact, despite the exchange of histones with protamine is essential for the maturation of sperm, a residual percentage of genome (5–15 %) retains the nucleosomal organization [109]. These retained nucleosomes play a key role in the contribution of the paternal genome in embryonic development [110]. In fact, the paternal DNA packaging in spermatozoa seems to have a potential role in transmitting an epigenetic profile to the zygote in early embryogenesis [111], since the nucleosome retention takes place in hypomethylated regions corresponding to the promoters of developmental transcription and signaling factors which are the target of transcription factors such as *OCT4*, *NANOG*, *SOX2*, and *KLF4* [110]. In this view, it is possible to hypothesize that an alteration of the correct distribution of nucleosome retention within sperm DNA could produce an impairment of embryo development. As a matter of fact, Hammoud et al. demonstrated the presence in infertile men of randomly distributed histone retention genome-wide, with alteration of the methylation status of candidate developmental promoters and imprinted loci [111]. Moreover, genes in histone-bound regions appear more susceptible to DNA damage induced by smoking, obesity, and aging as compared to protamine-bound regions, due to the incapacity of sperm to repair DNA damage [112]. Histone-bound regions play a crucial role in the activation of paternal genome transcription in the early embryo, since while paternal protamines are replaced by maternal histones in the first 4 to 6 h after fertilization, this does not occur for paternal histones, which are thus likely inherited by the embryo [113]. Thus, the reported data above suggest that, in some instances, epigenetic defects of the sperm could induce not only a poor sperm quality, but also a decreased ability of development of the generated embryo after the fusion of the gametes, providing a possible explanation for a number of early pregnancy loss after both in vivo and in vitro fertilization.

### Epigenetics and ART

A large amount of literature data, including human and animal studies, has raised concerns about an increased risk of different diseases in the offspring generated by the use of ART [114, 115]. Several evidences have suggested that the majority of these abnormal conditions are related to epigenetic alterations.

#### Data from ART in animal models

Early studies carried out in mice had showed that alterations affecting the development and growth of the fetuses were linked to ovulation induction, manipulation of eggs, or embryo culture in vitro [116–119]. A confirmation came from Khosla et al. showed that preimplantation mouse embryos cultured in the presence of serum can change the expression and methylation of several imprinted genes (such as *H19*, *IGF2*, *GRB10*, and *GRB7*) and that these aberrant epigenetic modifications lead to abnormal fetal growth in ART animals [120].

Moving to different animal models, Young et al. evidenced the presence of the “large offspring syndrome” (LOS) (large size at birth, increased birth weight, breathing difficulties, reluctance to suckle, and sudden perinatal death) in sheep and cattle derived from cultured embryo [121]. Subsequently, the pathogenesis of LOS was suggested to be associated with epigenetic abnormalities, leading to loss of imprinting and over-expression of *IGF2* receptor gene [122, 123]. Factors in the ART procedures, triggering these imprinting errors, were not clearly identified, but the onset of the syndrome appeared to be dependent by in vitro culture conditions [122, 123].

#### Data from ART in human

In human, a larger prevalence of syndromes related to imprinting alteration, particularly Beckwith-Wiedemann and Angelman syndromes, has been reported in children born after ART when compared to non-ART children [124–126]. Interestingly, the phenotype of Beckwith-Wiedemann syndrome, an overgrowth condition characterized by large size at birth, macroglossia, and visceromegaly, closely remembers the previously described LOS in cows and sheep generated by in vitro fertilization, suggesting a common mechanism of origin of these conditions. On the other hand, also a disproportionate number of low birth weight cases has been observed in ART children [127].

The association between ART and altered DNA epigenetic profiling has been demonstrated also by studies based on the analysis of the methylation status of CpG sites in the promoters of 700 genes of placenta and cord blood obtained from children conceived in vitro and in vivo [128]. This analysis showed hypomethylation of most CpG sites in the placenta and hypermethylation of most CpG sites in cord blood in the group of children conceived by in vitro fertilization as compared to naturally conceived children. Interestingly, the genes showing different expressions in the two groups appeared to be involved in chronic metabolic disorders including obesity, type II diabetes, and high blood pressure [128]. These data are in agreement with other studies reporting an increased risk of disturbs in body fat composition, changes in blood pressure, and increase in the late infancy growth velocity in children generated by ART procedures as compared to control children [129–131]. In addition, other studies showed a high risk of obesity or type II diabetes in adult life in children generated by ART [132]. These results strongly suggest that the effect of ART procedures could be manifested not only at birth, but also in late infancy or even in adult life.

As in animal models, the in vitro culture conditions have been suggested as a main cause of epigenetic defects in the offspring generated by ART also in human [133]. In addition, it has been proposed that the low birth weight observed after ART, as well as in small for gestational age and very premature children, is the result of an unfavorable embryonic, fetal, or neonatal environment, involving also epigenetic mechanisms, potentially related to metabolic alterations in late childhood [134]. However, several evidences have suggested that a crucial role could be also played by epigenetic defects in the sperms used in ART protocols. In fact, several authors suggested that DNA methylation changes at imprinted loci are inherited from the sperm of men with oligozoospermia [82, 135, 136]. This could suggest that the increase of DNA methylation variations in ART depends, at least in part, on the presence of epigenetic defects of male gamete. In this view, some authors have proposed the possible usefulness of analyzing the imprinting methylation status in the routine sperm examination for ART treatment [136]. Moreover, it has been hypothesized that the use of sperms with an abnormal P1/P2 ratio or defects of histone–protamine may be responsible of imprinting diseases in the offspring conceived with ART [50]. Unfortunately, this argument remains poorly understood, as demonstrated by other studies reporting that epigenetic abnormalities detected in sperms of oligozoospermic patients do not appear to be associated with ART outcome, suggesting that further studies are required in order to shed light on the relationship between sperm epimutation and alterations in children generated by ART [137].

### Environmental agents inducing epigenetic modifications

Several environmental and lifestyle factors (stress, physical activity, alcohol intake, smoke, shift work) are known to affect male and female fertility [138], and in many cases, they have been shown to influence the occurrence of epigenetic modifications with implications for human diseases [139]. The presence of an environmental epigenetic inheritance through gametes has been evidenced by studies carried out on different animal models [140]. A few studies have suggested that food or physical activity can influence histone modifications and miRNA expression. Dashwood et al. demonstrated that a single intake of cruciferous vegetables inhibits HDAC activity in mononuclear cells of peripheral blood promoting H3 and H4 acetylation [141], while other studies demonstrated that exposure to cigarette smoke causes a down-regulation of mir-34b, mir-421, mir450-b, mir-466, and mir-469 [142]. Anyway, the largest body of evidence comes from studies investigating the environmental effects on DNA methylation. Alterations in this process have been demonstrated to be induced in specific genome regions by toxic chemicals, high intake of alcohol and mother’s diet, or smoking during intrauterine life [143, 144]. Further information in this field has been provided by studies investigating the role played by paternal exposures to various pollutants and lifestyle-related conditions on the health status of the offspring and of the future generations.

#### Paternal exposure to toxins or ionizing radiation

Great attention has been devoted to the effects of paternal exposures to environmental toxins or low-dose ionizing radiation, and of paternal lifestyle [145]. Several studies had previously demonstrated the presence of a strong association between paternal occupational exposures to chemicals and harmful health outcomes in the offspring. Feychting at al. demonstrated an increased risk of nervous system tumors related to paternal occupational exposure to pesticides and of leukemia related to woodwork by fathers [146]. Reid et al. evidenced the presence of high exposure to exhausts by paternal grandmothers of children with acute lymphoblastic leukemia [147]. However, many of these conditions are likely related to the presence of mutations in sperm DNA, thus representing a genetic, rather than epigenetic, mechanism. Is there any evidence supporting the presence of epigenetic mechanism driving the effects to the offspring of the paternal exposure to chemicals? Once again, the most relevant data in support of this hypothesis come from studies on animal models, showing that male exposure to pesticides or other harmful chemicals can be responsible for defects in the gametes and abnormal development of the offspring mainly via altered DNA methylation patterns in the germ line [148, 149]. Anway et al. evidenced that a transient embryonic exposure to the endocrine disruptor vinclozolin during gonadal sex determination in rats produced several diseases affecting the prostate, kidney, immune system, testis, as well as different cancers in the subsequent generations, suggesting a potential transgenerational effect [148]. Similar results were obtained by Guerrero-Bosagna et al., who showed that transient exposure of the F0 generation gestating female to vinclozolin during gonadal sex determination caused adult onset disease in the F3 generation male and female mice [149].

Also, ionizing radiations have been recently invoked as a risk factor for alterations of DNA methylation. These radiations trigger a series of processes on the cells as genotoxic alterations including DNA breaks, but the actual mechanism leading to a transgenerational effect is still poorly understood. Dubrova et al. suggested an epigenetic mechanism of transmission of the radiation-exposure signal through sperm, likely involving DNA methylation and affecting DNA repair processes [150]. These authors suggested that the persistence of instability into the germ line of unexposed offspring of irradiated mice could be responsible of mosaicism in germ cells, a well-known mechanism in the origin of human genetic disorders [150].

More recently, it has been suggested that a crucial role in transgenerational radiation effects, such as genomic and epigenomic instability, could be played by the Piwi-interacting RNAs (piRNA) pathway, involved in the maintenance of genomic stability by facilitating DNA methylation of transposable elements and also implicated in other epigenetic alterations affecting a variety of cellular regulation processes [151]. Another experiment on animal models supports transgenerational epigenetic changes as a result of parental exposure to genotoxic stressors, as irradiation, nutrition, and intake of anti-androgen compounds [152]*.* For example, it has been demonstrated that treatment with the anti-androgen compound vinclozolin on female mice induces epigenetic effects in the sperm of their offspring as compared to controls [153]. This study highlighted an increased methylation of the differentially methylated domains (DMDs) of maternal *PEG1*, *PEG3*, and *SNRPN* genes and decreased methylation of paternal *H19* and *GTL2* genes.

#### Paternal diet

One of the most intriguing topics in the field of epigenetic modifications of the germ line is represented by the influence played by the paternal diet on gametogenesis. The first evidences of this association came from animal models. Carone et al. demonstrated that male mice fed with low-protein diet generated an offspring showing an increased expression of genes involved in the synthesis of lipids and cholesterol, as compared to the offspring of control male mice fed with a normal diet. Based on these results, authors suggested that cholesterol and lipid metabolism in an offspring can be strongly affected by paternal diet [154]. This study was carried out by a whole-genome characterization of cytosine methylation patterns and RNA content in sperm obtained from mice submitted to low-protein or caloric restriction diets and controls. Authors detected similar cytosine methylation patterns in all three conditions, thus suggesting that the sperm epigenome is largely unaffected by these diets and that changes in relatively few loci can have profound effects in the developing animal [154]. However, in a more recent study, Radford et al. demonstrated that in utero undernourishment perturbs the adult sperm methylome, suggesting that alterations in gamete methylation could induce alterations in chromatin architecture, transcriptional networks differentiation, or tissue structure, and in turn is able to contribute to the intergenerational transmission of environmentally induced diseases [155].

In another animal study, Ng et al. showed the presence of pancreatic alterations, with early onset impaired insulin secretion and glucose tolerance worsening with time, in the female offspring of male mice fed with a high-fat diet [156]. This effect was mediated by the altered expression in adult female offspring of 642 pancreatic islet genes, belonging to 13 functional clusters, including cation and ATP binding, and cytoskeleton and intracellular transport. Fullston et al. demonstrated the presence of altered global methylation in mature sperm and abnormal testis transcription of male mice consuming a high-fat diet, with metabolic disturbances in the next generations [157]. In addition to experimental data on animal models, very interesting data about the role played by diet on the epigenetic modifications and on the consequent transgenerational effects are available in human as well. During the winter of 1944–45 of World War II, in the Netherlands, as a reprisal against the activity of the Dutch government-in-exile aimed to disrupt the transport of German reinforcements and troops, the Germans banned all food and fuel transports to Netherlands, inducing a severe famine, with the official daily rations for the general adult population decreasing gradually from about 1800 calories (December 1943) to below 800 calories (April 1945). The situation improved in a very short time after the liberation of the Netherlands on May 1945, with the rations raising up to over 2000 calories a day by June 1945 [158]. The famine caused a severe mortality in the population of Amsterdam, but nevertheless, several babies were conceived and birthed during that period. Several decades later, a number of studies investigated the health status of people born in Amsterdam during the famine period in order to shed light on the effects of malnutrition on the health of the offspring in adult life. In a first time, these studies evidenced an association with chronic diseases in adult life in the offspring (coronary heart disease, atherogenic lipid profile, obesity, raised levels of plasma fibrinogen, and decreased levels of factor VII), strongly related to the timing in gestation of exposure to famine [158, 159]. However, by analyzing the results of these epidemiological studies with the aid of molecular tools, it has become clear that the Dutch famine families study has provided the first direct evidence for epigenetic programming through prenatal famine exposure. In fact, it was clearly demonstrated that periconceptional exposure to famine produced an under-methylation (likely related to a deficiency in methyl donors) in the differentially methylated region (DMR) of the maternally imprinted *IGF2* gene [160], suggesting that early undernutrition can cause epigenetic changes persisting throughout life. On the other hand, there was no variation in *IGF2* methylation status in individuals exposed to famine in later gestation. Further studies evidenced that persistent changes in DNA methylation represent a common consequence of prenatal famine exposure and that they can be affected by the sex of the exposed individual and the gestational timing of the exposure [161]. More recently, it has been demonstrated that prenatal malnutrition-associated DMRs (P-DMRs) mostly occur in regulatory regions of genes showing differential expression during early development [162].

All the above-reported studies suggest that undernutrition plays a direct effect on the fetus during the early phases of development. However, why should we exclude that the target of the famine could be represented also by gametes other than by embryo? Several evidences strongly suggest a role played by paternal diet on the healthy status of children, as confirmed by the above-described results on animal models. In human, the link between grandparental nutrition and grandchild’s growth was at first examined by Bygren et al., who demonstrated that a surfeit of food in the environment when the paternal grandfather was a boy was related to a shortening of the proband survival [163]. The same group subsequently demonstrated that a limited availability of food during the father’s prepuberal age was related to a low cardiovascular disease mortality of the proband, while paternal grandfather exposure to a surfeit of food during the same period was related to increased diabetes mortality in the proband [164], suggesting epigenetic inheritance as a strong candidate for these phenomena [165]. Soubry et al. demonstrated the presence of alterations in the methylation status at multiple imprint regulatory regions in children with obese parents, suggesting a preconceptional influence of parental lifestyle and nutrition on the programming of imprint marks during gametogenesis [166]. In particular, the significant association between paternal obesity and altered methylation in the offspring suggests the susceptibility of the developing sperm to environmental insults. Very recently, the evidence of a role played by changes in paternal grandmothers’ early food supply on the risk of cardiovascular mortality of the female grandchildren have also suggested an X-linked epigenetic inheritance via spermatozoa [167]. However, it has also been stressed that a true transgenerational inheritance in response to diet should be examined on the third generation, which represents the actual first “unexposed” one, being the first filial generation directly exposed to the maternal diet, and deriving the second from gametes exposed in utero [168]. Despite these limitations, the possible presence of transgenerational effects related to the epigenetic effect of paternal diet remains a very interesting topic.

#### The four windows of epigenetic susceptibility

A crucial question concerning the role played by environmental agents in the epigenetic modifications of the male gamete is the following: when are the effects of such exposures transferred to the male gamete? Soubry et al. identified four potential windows of susceptibility during the development of the paternal germ line and zygote [145]. The first window is represented by paternal embryonic development, when PGC undergo genome-wide epigenetic erasure during migration to the genital ridge. Defects in this process, as well as in the maintenance of some protected regions, could be caused by internal or external factors during early development. The second window is represented by paternal prepuberty, since in this period, de novo methylation at imprinted gene loci occurs. The third window can be identified in the period in which spermatogenesis, and in particular the development from spermatogonium to spermatocytes, occurs, since methylation patterns are established during this time. This window appears to be a very important one, representing the reproductive period of the subject, when a careful evaluation of his lifestyle, with prevention of environmental stressors, could be used as a preventive strategy. Finally, the fourth window is represented by the periconception period and the zygote stage, when histone retention in certain genes could represent a potential mechanism for inheritance of environmentally induced epigenetic marks. While studies investigating the effect of environmental agents in the latter three windows are currently carried out, mostly on animal models but also in humans, it is more difficult to investigate epigenetic modifications in PGCs. However, the recently reported discovery that amniotic fluid stem cells (AFSCs) share a number of features with PGCs [169] provides a novel cellular model for the study of the effect played by environmental agents in altering the epigenetic processes occurring in these cells, opening new scenarios in this field of study.

Finally, it must be stressed that aging represents another possible risk factor for epigenetic modifications increasing the risk of neuropsychiatric disorders such as autism and schizophrenia. Jenkins et al. have recently identified 139 regions significantly and consistently hypomethylated and 8 regions significantly hypermethylated with aging, with a total of 117 genes involved [170]. These authors evidenced that a portion of the age-related changes in sperm DNA methylation involves genes previously associated with schizophrenia and bipolar disorder.

## Conclusions

**Fig. 2:**
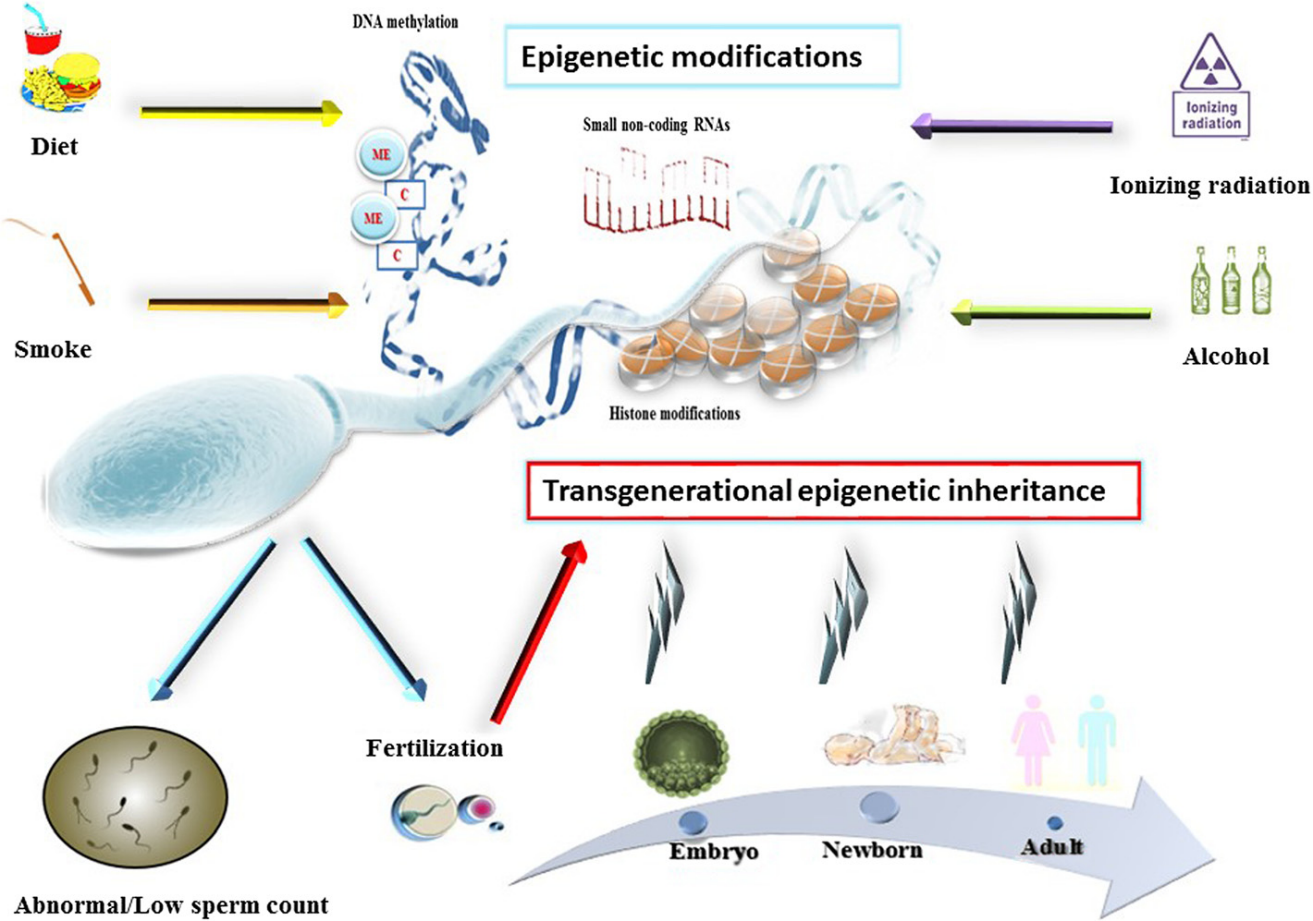
Epigenetic alterations induced by lifestyle and environmental factors (diet, smoking, radiation, alcohol consumption, etc.) can have substantial effects on the sperm function. As a first consequence, these modifications can induce sperm alterations leading to impairment of male fertility. When fertilization occurs, spontaneously or by ART, transgenerational epigenetic effects can be observed, in details leading to (1) alterations of embryo development, (2) congenital diseases at birth, and (3) late onset diseases (obesity, hypertension, diabetes, etc.) in the adult life

As evidenced by the large amount of studies carried out in this field, epigenetic mechanisms play a key role in the proper function of the male gamete, and alterations in these mechanisms can widely affect human reproduction. The effect of epigenetic modification of sperm gene function can affect the reproductive outcome in at least four different levels: (1) impairment of male fertility due to alterations in sperm number and morphology; (2) alterations of embryo development; (3) poor outcome of the ART protocols; and (4) risk of pathologies in the adultness for the offspring (Fig. 2). Due to the huge interest devoted to this topic by the scientific community, related to the possible implications in the field of human reproduction and health, our knowledge about the above-discussed mechanisms are increasing day by day. Recent studies have highlighted the molecular mechanisms underlying the epigenetic transgenerational inheritance of some disease. Guerrero-Bosagna et al. have demonstrated the presence of unique consensus DNA sequence motifs, zinc finger motifs, and G-quadruplex sequences in transgenerational DMR in sperm, which, by the interaction of molecular factors, could induce alterations of the chromatin structure and accessibility of proteins with DNA methyltransferases altering de novo DNA methylation patterns [171].

It can be suggested that in the next future, the study of epigenetics and epigenomics will likely represent a crucial step in the diagnostic workup of the infertile male, especially in cases submitted to ART, where it will be necessary to select adequately functional sperm to avoid the epigenetic alteration impact on the procedure. So far, the application of the analysis of epimutations in the male gamete in the clinical practice is hampered by the lack of complete information about the involved genes and by the use of expensive, low-throughput techniques. However, due to the large number of ongoing studies in this field, a clearer picture of the situation should be available in a short time, and the set-up of specific assays will likely reduce the costs and the time of these analyses. Further information will likely be provided by studies investigating the role played by sperm non-coding RNA in male fertility, which represents a very promising field of study [172]. Another very exciting field is represented by the potential role played by the non-sperm fraction of the seminal fluid, since postejaculatory effects on sperm survival and functional competence have been reported [173]. Surprisingly, seminal plasma may affect offspring independently of sperm, by stimulating the production of embryotrophic cytokines and growth factors by the female reproductive tract [173]. The alteration of this process induces abnormal fat deposition and metabolic phenotype in the offspring, particularly in the males [174]. Finally, great attention should be devoted to the role played by environmental agents both in determining and in repairing epigenetic alterations. In fact, the identification of the specific doses and times of action of agents able to induce epigenetic alteration of sperm DNA or to restore the functional conditions will be likely of great help in the treatment of spermatogenetic defects and/or poor outcome of both normal and in vitro fertilization. Most importantly, the possibility that paternal lifestyle could affect the health of the offspring during lifetime opens a novel and exacting scenario in the prevention of common, late onset diseases [175].

## Abbreviations

5hmC: 5-hydroxymethylcytosine
AFSCs: amniotic fluid stem cells
ART: assisted reproduction techniques
CNVs: copy number variants
COBRA: combined bisulfite restriction analysis
DMDs: differentially methylated domains
DMR: differentially methylated region
DNMTs: DNA methyltransferases
ESCs: embryonic stem cells
H3-K4: histone H3 lysine 4
H3-K9: histone H3 lysine 9
HAT: histone acetyl transferase
HDAC: histone deacetylase
HDM: histone demethylase
HMT: histone methyltransferase
ICR1: imprinting control region 1
lincRNAs: large intergenic non-coding RNAs
LOS: large offspring syndrome
NGS: next-generation sequencing
NZ: normozoospermic
OAT: oligoasthenoteratozoospermic
P-DMRs: prenatal malnutrition-associated differentially methylated regions
PGCs: primordial germ cells
piRNA: Piwi-interacting RNAs
TP: transition proteins
UPD: uniparental disomy
WGBS: whole-genome bisulfite sequencing
